# Editorial: Advances in Virtual Agents and Affective Computing for the Understanding and Remediation of Social Cognitive Disorders

**DOI:** 10.3389/fnhum.2015.00697

**Published:** 2016-01-07

**Authors:** Eric Brunet-Gouet, Ali Oker, Jean-Claude Martin, Ouriel Grynszpan, Philip L. Jackson

**Affiliations:** ^1^Faculté de Médecine, Université Versailles Saint-QuentinVersailles, France; ^2^Pôle de Psychiatrie, Centre Hospitalier de VersaillesLe Chesnay, France; ^3^Laboratoire d'Informatique pour la Mécanique et les Sciences de l'Ingénieur-Centre National de la Recherche ScientifiqueOrsay, France; ^4^Institut des Systèmes Intelligents et de Robotique, Centre National de la Recherche Scientifique, Université Pierre et Marie CurieParis, France; ^5^École de Psychologie, Université LavalQuébec, QC, Canada

**Keywords:** virtual reality, social cognition, affective computing, psychotherapy, social cognition in clinical groups

The present topic emphasizes the parallel development of concepts in social neurosciences and in other domains such as computer science, affective computing, virtual reality development, and even hardware technologies. While several researchers in neurosciences pointed out the necessity to consider naturalistic social cognition (Zaki and Ochsner, 2009), the second person perspective (Schilbach et al., 2013), social interaction (Pfeiffer et al.) and reciprocity (de Bruin et al.), both computer and software developments allowed more and more realistic real-time models of our environment and of virtual humans capable of some interaction with users. A new convergence between scientific disciplines might occur from which it is tricky to predict the outcomes in terms of new concepts, methods, and uses.

Although this convergence meets ongoing societal changes (increasing social demands on computer technologies, augmenting funding), it comes with several difficulties for which the current Frontiers in' topic strives to bring some positive answers, and to provide both theoretical arguments and experimental examples. The first problem was about concepts and vocabulary as contributions described in the following were authored by neuroscientists, computer scientists, psychopathologists, each coming with separate knowledge, and key literature. A special attention was given to avoid purely technical descriptions and to focus on the added value of virtual reality in neurosciences and psychopathology. Another problem concerned methods: more complex computerized interaction models results in unpredictable and poorly controlled experiments. In other words, the assets of naturalistic paradigms may be alleviated by the difficulty to match results between subjects, populations, and conditions. It is crucial to consider this question when investigating pathologies that are associated with profoundly divergent behavioral patterns. The last issue we encountered was about heterogeneity of the objectives of the researches presented here. While selection criteria focused on the use of innovative technologies to assess or improve social cognition, the fields of application of this approach were quite unexpected.

The first group of contributions exemplifies how innovation in methods improves understanding and assessment of social cognition disorders or pathology. Timmermans and Schilbach provide technological orientations for investigating alterations of social interaction in psychiatric disorders by the use of dual interactive eye tracking with virtual anthropomorphic avatars. Joyal et al. bring concurrent and construct validities of a newly developed set of virtual faces expressing six fundamental emotions. The relevance of virtual reality was shown with two contributions focusing on anxiety related phenomena. Jackson et al. describe a new environment allowing investigating empathy for dynamic FACS-coded facial expressions including pain. Based on a systematic investigation of the impact of social stimuli modalities (visual, auditory), Ruch and collaborators are able to characterize the specificity of the interpretation of laughter in people with gelotophobia (Ruch et al.). On the related issue of social anxiety, Aymerich-Franch et al. presented two studies in which public speaking anxiety has been correlated with avatars' similarity of participants' self-representations. Finally, three contributions demonstrate the feasibility and the usefulness of virtual reality settings on chronic developmental and psychiatric conditions like high functioning autism (HFA) or schizophrenia. Grynszpan and Nadel present an original eye-tracking method to reveal the link between gaze patterns and pragmatic abilities in HFA. About schizophrenia, Oker et al. discuss and report some insights on how affective and reactive virtual agents might be useful to assess and remediate several defects of social cognitive disorders. About assessment within virtual avatars on schizophrenia, Park et al. focused on effect of perceived intimacy on social decision making with schizophrenia patients.

The second set of contributions focus on therapeutic intervention with either a cognitive behavioral therapy (CBT) framework or with cognitive remediation/training procedures. CBT interventions share common principles and take in consideration thoughts, feelings or emotions and behaviors in order to generate gradual modification of each of these levels thanks to thought and schema analysis, stress reduction procedures, etc. They were observed to be somehow useful for the treatment of depression, stress disorders, phobias, and are gaining some authority in personality disorders and addictions. The main asset of new technologies is the possibility to control the characteristics of symptom-eliciting stimuli/situations, and more precisely the degree to which immersion is enforced. For example, Baus and Bouchard provide a review on the extension of virtual reality exposure-based therapy toward recently described augmented reality exposure-based therapy in individuals with phobias. Concerning substance dependence disorders, Hone-Blanchet et al. present another review on how virtual reality can be an asset for both therapy and craving assessment stressing out the possibilities to simulate social interactions associated with drug seeking behaviors and even peers' pressure to consume.

The last contributions of the topic concern social cognitive training or remediation in severe and chronic mental disorders (autistic disorders, schizophrenia). Here, therapies are based on drill and practice or strategy shaping procedures, and, most of the time, share an errorless learning of repeated cognitive challenges. Computerized methods were early proposed for that they do, effortlessly and with limited costs, repetitive stimulations. While, repetition was incompatible with realism in the social cognitive domain, recent advances provide both immersion and full control over stimuli. Georgescu et al. exhaustively reviews the use of virtual characters to assess and train non-verbal communication in HFA. Regarding schizophrenia remediation, Peyroux and Franck presented a new method, named RC2S, which is a cognitive remediation program to improve social cognition in schizophrenia and related disorders.

To conclude briefly, while it is largely acknowledged that social interaction can be studied as a topic of its own, all the contributions demonstrate the added value of expressive virtual agents and affective computing techniques for the experimentation. It also appears that the use of virtual reality is at the very beginning of a new scientific endeavor in cognitive sciences and medicine.

## Funding

Authors EB, AO, JM received funding from ANR ANR-11-EMCO-0007.

### Conflict of interest statement

The authors declare that the research was conducted in the absence of any commercial or financial relationships that could be construed as a potential conflict of interest.
